# The application of deep learning based diagnostic system to cervical squamous intraepithelial lesions recognition in colposcopy images

**DOI:** 10.1038/s41598-020-68252-3

**Published:** 2020-07-15

**Authors:** Chunnv Yuan, Yeli Yao, Bei Cheng, Yifan Cheng, Ying Li, Yang Li, Xuechen Liu, Xiaodong Cheng, Xing Xie, Jian Wu, Xinyu Wang, Weiguo Lu

**Affiliations:** 1 Women’s Reproductive Health Laboratory of Zhejiang Province, Women’s Hospital, School of Medicine, Zhejiang University, Hangzhou, 310006 Zhejiang China; 2 Department of Gynecologic Oncology, Women’s Hospital, School of Medicine, Zhejiang University, Hangzhou, 310006 China; 3 Center for Uterine Cancer Diagnosis & Therapy Research of Zhejiang Province, Hangzhou, 310006 China; 4 College of Computer Science and Technology, Zhejiang University, Hangzhou, 310027 China

**Keywords:** Imaging, Cancer, Health care

## Abstract

*Background* Deep learning has presented considerable potential and is gaining more importance in computer assisted diagnosis. As the gold standard for pathologically diagnosing cervical intraepithelial lesions and invasive cervical cancer, colposcopy-guided biopsy faces challenges in improving accuracy and efficiency worldwide, especially in developing countries. To ease the heavy burden of cervical cancer screening, it is urgent to establish a scientific, accurate and efficient method for assisting diagnosis and biopsy. *Methods* The data were collected to establish three deep-learning-based models. For every case, one saline image, one acetic image, one iodine image and the corresponding clinical information, including age, the results of human papillomavirus testing and cytology, type of transformation zone, and pathologic diagnosis, were collected. The dataset was proportionally divided into three subsets including the training set, the test set and the validation set, at a ratio of 8:1:1. The validation set was used to evaluate model performance. After model establishment, an independent dataset of high-definition images was collected to further evaluate the model performance. In addition, the comparison of diagnostic accuracy between colposcopists and models weas performed. *Results* The sensitivity, specificity and accuracy of the classification model to differentiate negative cases from positive cases were 85.38%, 82.62% and 84.10% respectively, with an AUC of 0.93. The recall and DICE of the segmentation model to segment suspicious lesions in acetic images were 84.73% and 61.64%, with an average accuracy of 95.59%. Furthermore, 84.67% of high-grade lesions were detected by the acetic detection model. Compared to colposcopists, the diagnostic system performed better in ordinary colposcopy images but slightly unsatisfactory in high-definition images. *Implications* The deep learning-based diagnostic system could help assist colposcopy diagnosis and biopsy for HSILs.

## Introduction

Ranking as the second most common cancer in the female reproductive system, cervical cancer still manifests high morbidity and mortality in developing countries including China, imposing a strong impact on the body health and quality of life of women^1^. Fortunately, studies have demonstrated that high-risk human papillomavirus (HPV) infection can be the definite etiology of cervical cancer^2–5^, making it possible to detect 80.7–98.7% of cervical intraepithelial neoplasia (CIN) early through screening combined with HPV testing and cytology^6–8^. As the gold standard for diagnosing cervical cancer and its precancerous lesions, colposcopy-guided biopsies play a key role in the early detection of cervical cancer.

In clinical practice, many factors affect the accuracy of cervical biopsies including the experience of the colposcopist, the location, size and depth of the lesions, and the menstrual status of the woman. Even by experienced colposcopists, the sensitivity of colposcopy varies from 81.4% to 95.7% to detect CIN, with a specificity of 34.2% to 69%^9–13^. Improving the accuracy of colposcopy is a key issue in the management of CIN.

Based on artificial intelligence and deep learning, computer assisted medical diagnosis can efficiently and scientifically deal with a large quantity of clinical data and achieve comparable performance on various medical tasks. Studies have suggested that medical artificial intelligence and computer assisted diagnosis can help detect lesions and improve diagnosis accuracy by using deep learning and medical image processing technology plus some possible physiological and pathological knowledge^14–16^. Studies in optical coherence tomography^17^, radiology^18^, computerized tomography scan^19^, colonoscopy^20^ and pathologic slides^15^ have indicated that computer algorithms can approach and even surpass the diagnostic accuracy of clinicians after training a large number of medical images in a convolutional neural network (CNN).

Early in 2009, Acosta et al. ^21^ used K-NN algorithm to automatically distinguish normal and abnormal cervical tissue in aceto-white pattern, and gained a sensitivity of 71% and the specificity of 59%. Years later, Asiedu et al.^22^ achieved the sensitivity, specificity, and accuracy of 81.3%, 78.6%, and 80.0% to distinguish CIN+ and benign tissues apart. Liming Hu et al.^23^ established a cohort and followed up for 7 years, using images shot by cervicography, to train and validate deep learning algorithm and gained higher accuracy compared to pap smear. Besides, Bing Bai et al.^24^ used K-means algorithm to automatically segment cervical region, indicating that cervical segmentation was feasible.

In all previous studies, only cervical acetic acid images were collected for training and validation. In the present study, we collected a quantity of both acetic images and iodine images with clinical information, and utilized them to train three models to separately classify, segment cervical squamous intraepithelial lesions (SILs) and detect high-grade squamous intraepithelial lesions (HSILs) to assist colposcopy-guided biopsy. Furthermore, an independent dataset of cases with high-definition colposcopy images was collected as a whole to evaluate the accuracy of the models for the second time. The performance of the models in the two datasets was compared with that of clinical colposcopists. The aim of the study was to establish a novel colposcopy diagnostic system to efficiently and accurately recognize and detect HSIL in colposcopy images and to assist colposcopists in diagnosis and biopsy.

## Results

### The basic information of the modelling dataset

**Figure 1:**
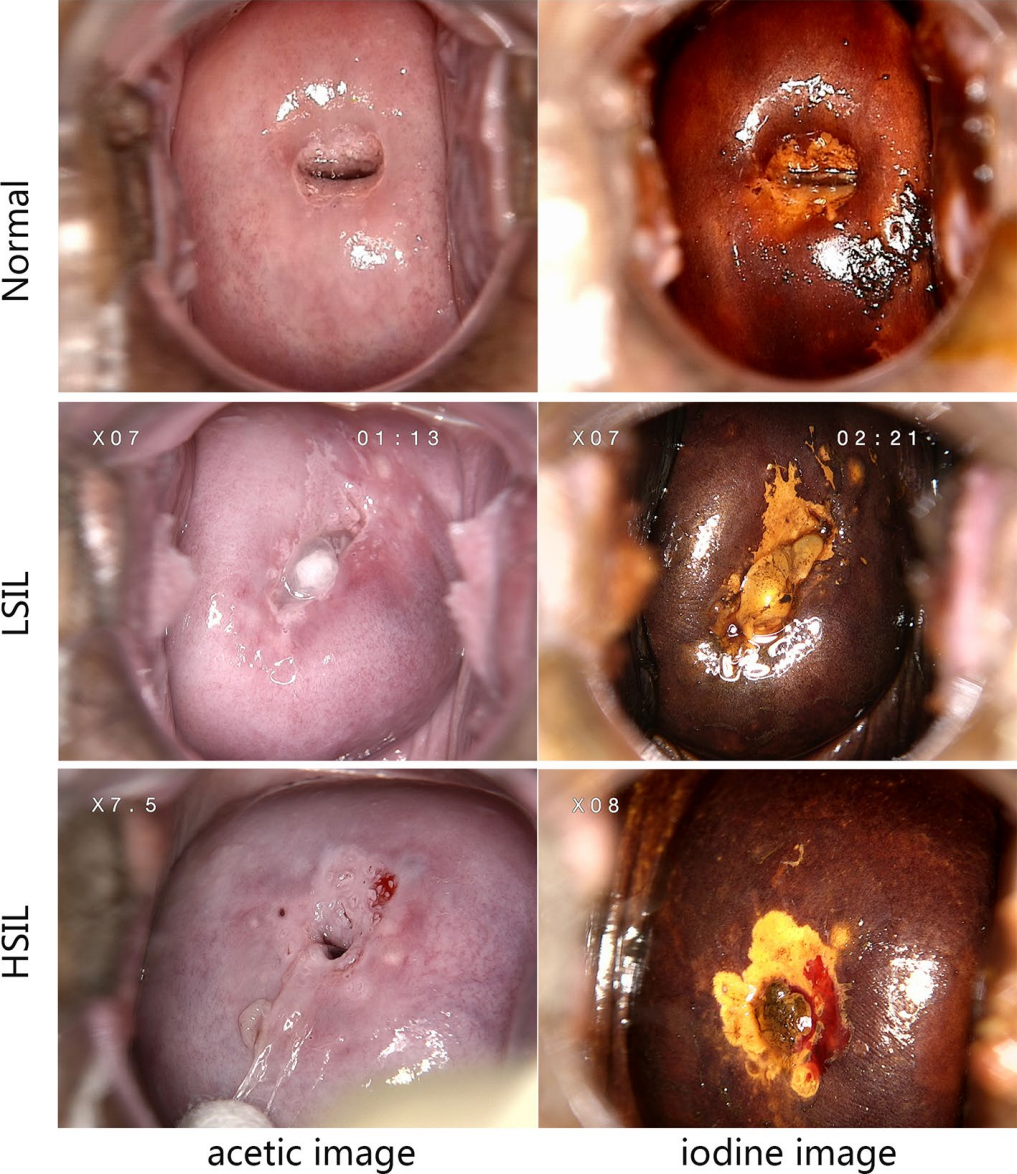
The representative acetic and iodine images of the normal, LSIL and HSIL case.

**Figure 2:**
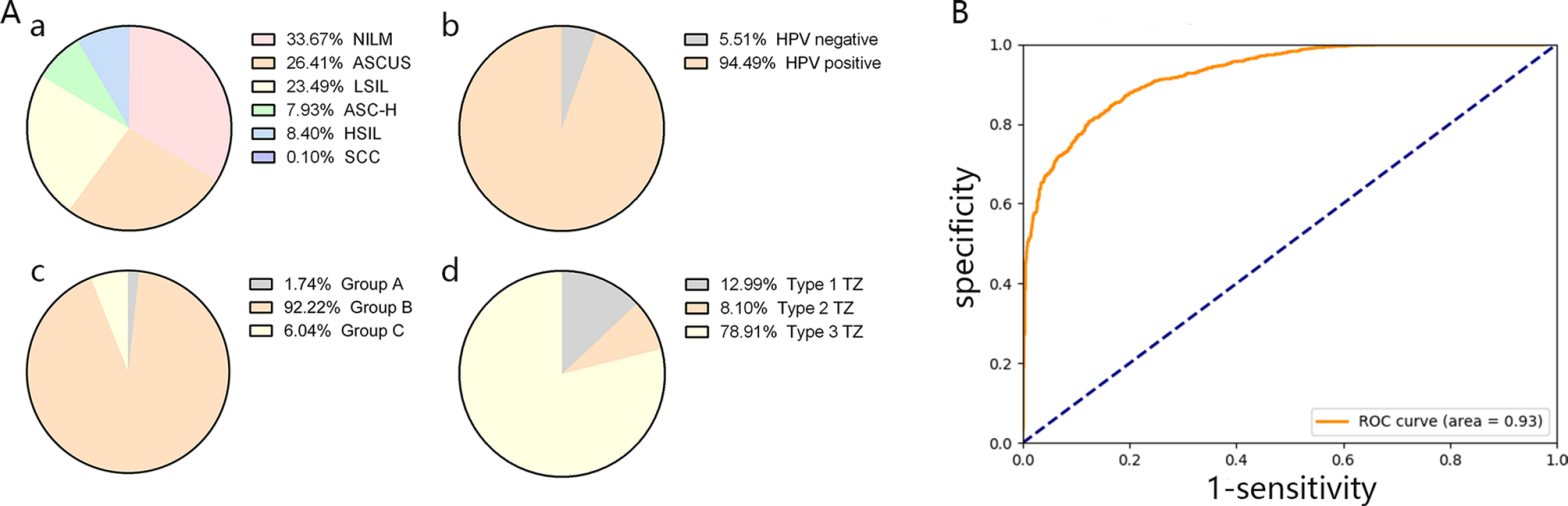
(**A**) a. The cytology distribution of the modeling dataset used in the research. b. The HPV status distribution of the modeling dataset used in the research. c. The age distribution of the modeling dataset used in the research. d. The TZ type distribution of the modeling dataset used in the research. (**B**) The ROC curve of the validation set of the modeling dataset using the classification model.

After enrolment, 22,330 cases were selected for model training and evaluation including 10,365 normal cases, 6,357 LSIL cases and 5,608 HSIL cases. Representative images of normal cases, LSIL cases and HSIL cases are presented in Fig. 1. The distributions of age, HPV infection status, cytology results and TZ type are presented in Fig. 2A.

### The ResNet model can simply classify colposcopy images into two categories

In the classification model, one acetic image, one iodine image, age, HPV testing result, cytology result and TZ type were used as input indices. Pathology diagnoses were used as the output indices. Of those, 10,365 normal cases, 6,357 LSIL cases and 5,608 HSIL cases were proportionally divided into the training set, the test set and the validation set at a ratio of 8:1:1. The final result of the valid set is listed in Table 1.

**Table 1:** The prediction result of the classification model in valid set.

Pathology prediction	Negative*	Positive*	Total
Negative*	856	175	1,031
Positive *	180	1,022	1,202
Total	1,036	1,197	2,233

*Negative represents the pathologic normal cervix. Positive represents the pathologic results of LSIL + .

The area under the curve (AUC) of the classification model reached 0.93 in the validation set (Fig. 2B), presenting a sensitivity of 85.38%, a specificity of 82.62% and an accuracy of 84.10%. In addition, the positive predictive value (PPV) and the negative predictive value (NPV) of the model were 85.02% and 83.03% respectively.

### The U-Net model can precisely segment the lesions in the cervix

**Figure 3:**
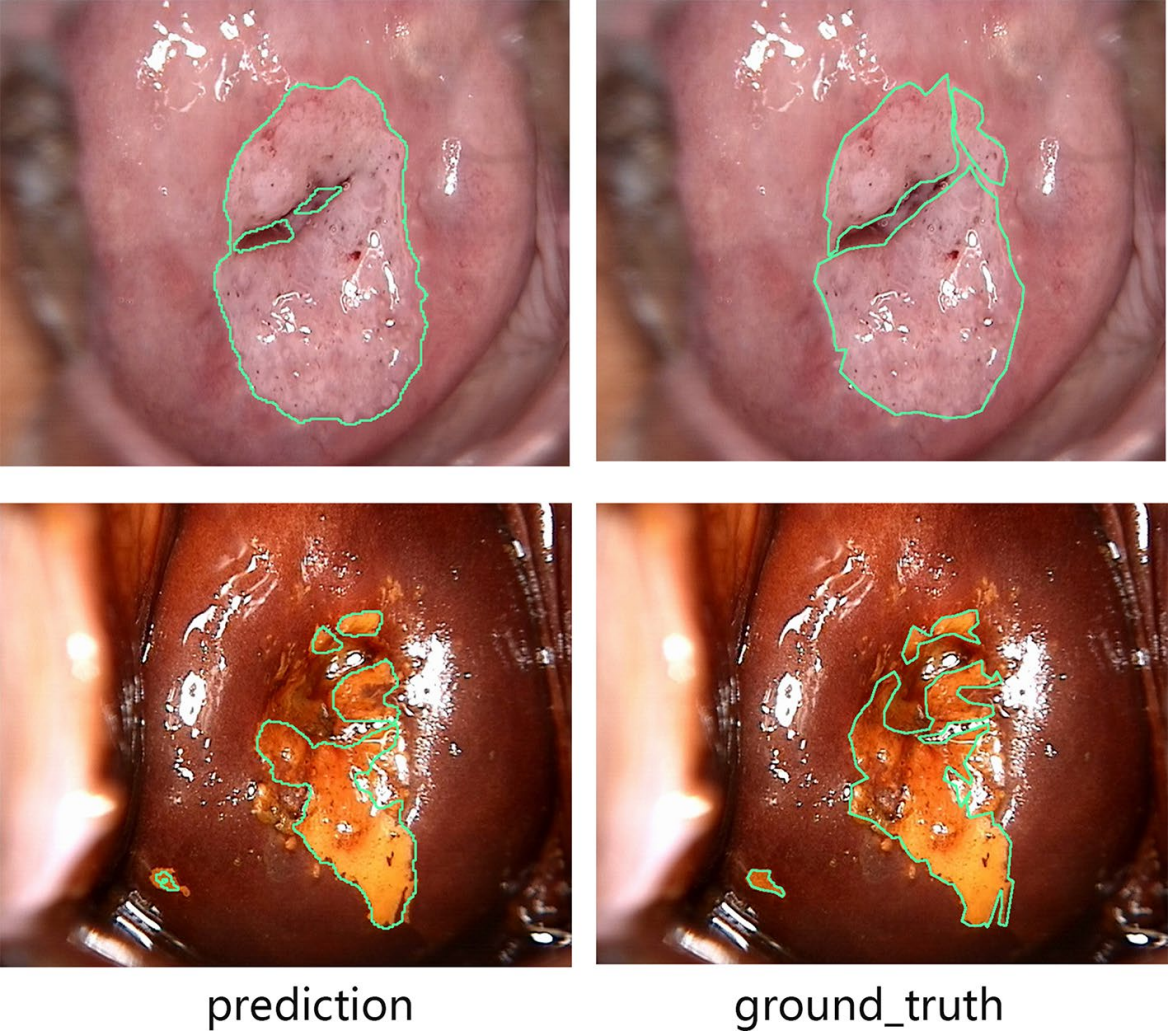
The representative the prediction (left) and groud truth (right) of the valid set using the acetic and iodine segmentation model.

In total, 11,198 acetic images and 11,198 iodine images were separately input into the segmentation model. Since the U-Net model was trained at the pixel level after annotation, the segmentation model outputs a prediction area consisting of pixels that may possibly be the SIL at the end. Figure 3 presents the ground truth area (right) and the predicted area (left) in both acetic images and iodine images. The representative results showed high consistency between the two areas. Representative failed images were shown in Fig. S1. Most of the missed lesions were finally pathologically diagnosed as LSIL, and the reason of misdiagnosed lesions was not clear.

In acetic images, the recalls of normal pixels and SIL pixels were 96.14% and 84.73%, respectively, and the DICE of normal pixels and SIL pixels were 97.53% and 61.64% respectively. The average accuracy of the U-Net model in acetic images was 95.59%. The results in the iodine model were almost the same. The recalls of normal and SIL pixels were 96.03% and 87.78% respectively, and the DICE of normal and SIL pixels were 97.58% and 63.80% respectively, with a total accuracy of 95.70% in iodine images.

### The MASK R-CNN model can detect HSIL lesion

**Figure 4:**
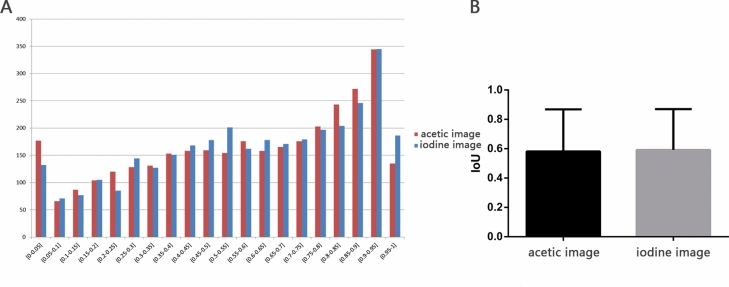
(**A**) The distribution of IoU in detection model in the valid set of ordinary images. (**B**) The mean IoU in detection model in the valid set of ordinary images.

**Figure 5:**
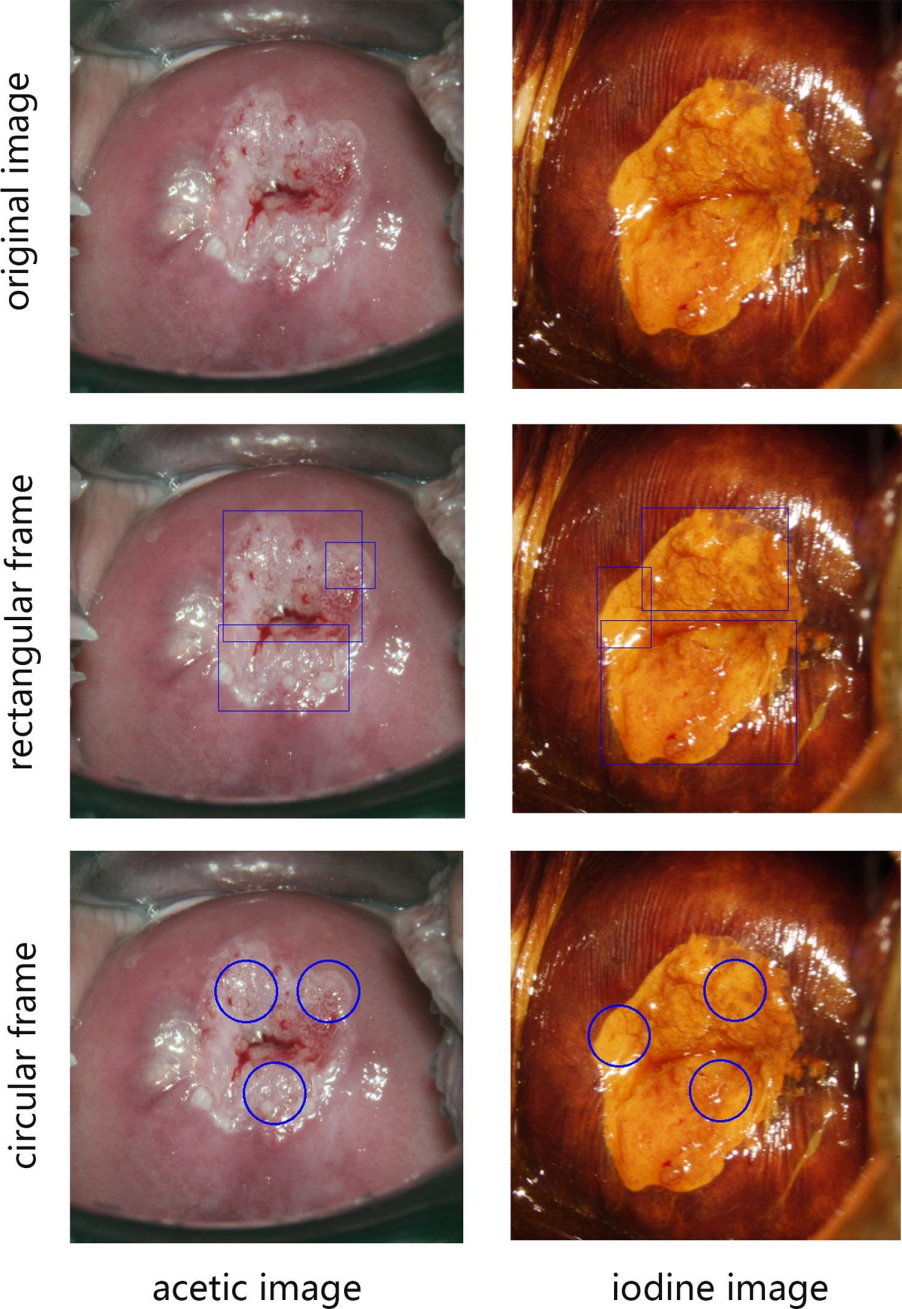
The representative original image, rectangle prediction frame, and circular prediction frame of the acetic image (left) and the iodine image (right) of the valid set using the detection model.

In total, 22,396 images of 11,198 cases were utilized in the detection model. Nevertheless, the acetic images and the iodine images were separately trained. Finally, several rectangular prediction frames are presented with the confidence coefficient to be HSIL. The distribution of IoU and the mean IoU of prediction frames in acetic images and in iodine images were shown in Fig. 4. To control the biopsy number, only the top 3 confidence HSIL prediction frames were adopted as the final results. More specifically, circular labels of fixed diameter were utilized to mark the most suspicious area to assist biopsy (Fig. 5). Representative failed images were shown in Fig. S2.

The results of the validation set with 1,120 cases are listed in Table 2 (in acetic images) and Table 3 (in iodine images). The recalls to detect HSIL in acetic images and in iodine images were 84.67% and 84.75%, respectively. The PPV for HSIL was 19.98% in acetic images and 21.22% in iodine images. Putting LSIL and HSIL together, the detection model can recall 82.55% and 82.45% of SIL in acetic mages and iodine images respectively, with a PPV for SIL of 62.09% and 64.41%.

**Table 2:** The prediction of HSIL in the detection model in acetic images.

Pathology prediction	Normal	LSIL	HSIL	Total
Normal	0	297	113	410
LSIL	0	0	0	0
HSIL	1,184	1,315	624	3,123
Total	1,184	1,612	737	3,533

**Table 3:** The prediction of HSIL in the detection model in iodine images.

Pathology prediction	Normal	LSIL	HSIL	Total
Normal	0	311	120	431
LSIL	0	0	0	0
HSIL	1,119	1,358	667	3,144
Total	1,119	1,669	787	3,575

At the patient level, the HSIL cases were regarded as “hit” when at least one HSIL lesion was accurately predicted. The detection model could “hit” 439 HSIL cases in all 503 HSIL cases in the validation set through acetic images and could “hit” 445 HSIL cases through iodine images. The sensitivity to predict HSIL cases was 87.27% and 88.47%, respectively.

### The validation results in high-definition images

**Figure 6:**
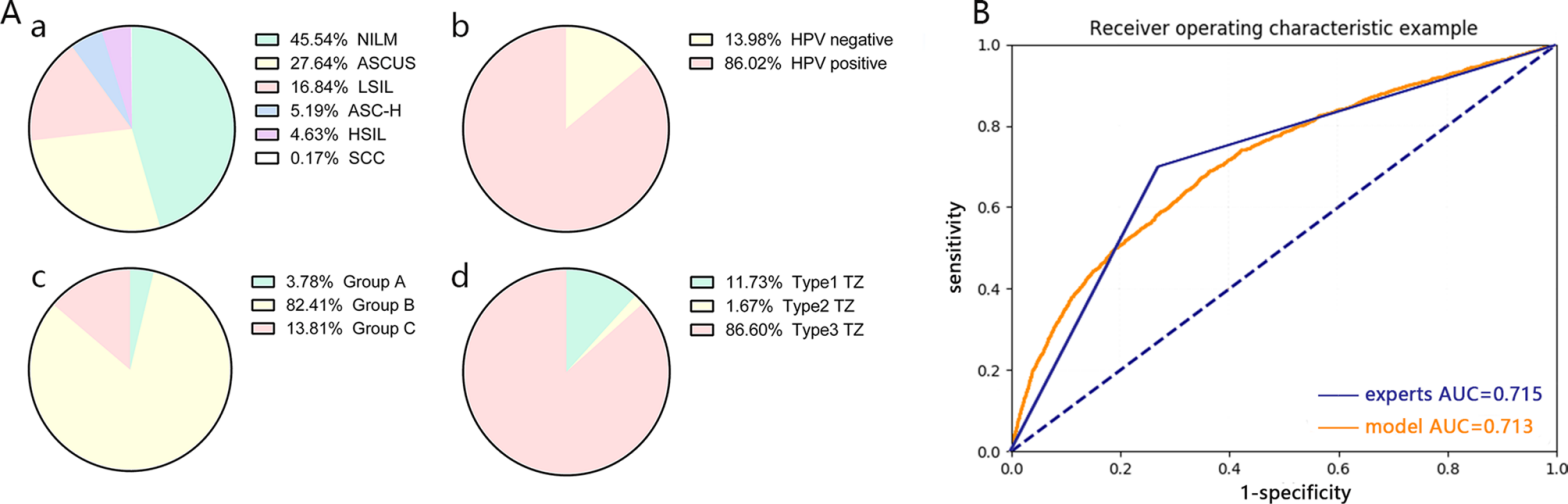
(**A**) a. The cytology distribution of the validation dataset used in the research. b. The HPV status distribution of the validation dataset used in the research. c. The age distribution of the validation dataset used in the research. d. The TZ type distribution of the validation dataset used in the research. (**B**) The ROC curve of the validation dataset using the classification model and the ROC curve of the colposcopists.

After selection, 5,384 cases were enrolled in the independent dataset from a total of 9,060 cases. All the images were shot by a high-definition electronic colposcope including 3,375 normal cases, 1,246 LSIL cases and 763 HSIL cases. The distributions of age, the results of HPV testing and cytology, and the TZ types are presented in Fig. 6A.

In the classification model, the sensitivity, specificity and accuracy in differentiating positive cases and negative cases in high-definition images were 73.37%, 58.16%, and 63.83%, respectively (Table 4). The PPV and NPV were 51.07% and 78.58%, respectively, with an AUC of 0.7127 (Fig. 6B). As a comparison, the sensitivity, specificity and accuracy of five colposcopy experts in women’s hospital, school of medicine, Zhejiang University to differentiate positive cases and negative cases were calculated and are presented in Table 5. The ROC curve of the experts was shown in Fig. 6B with an AUC of 0.715. Expert 1 and expert 2 were senior colposcopists with more than 10 years of experience, expert 3 and expert 4 were intermediate colposcopists who had almost 5 years of experience in colposcopy, and expert 5 was a senior colposcopist with 1 year of experience in colposcopy. From Table 5, we concluded that the performance of the classification model in ordinary images was much better than that of all five colposcopists, while the performance in high-definition images was comparable to that of intermediate and junior colposcopists. And the AUC of the experts were almost the same as that of the classification model in high-definition images, and were lower than that of the classification model in ordinary images.

**Table 4:** The prediction result of the classification model in validation dataset.

Pathology prediction	Negative*	Positive*	Total
Negative*	1963	535	2,498
Positive*	1,412	1,474	2,886
Total	3,375	2,009	5,384

*Negative represents the pathologic normal cervix. Positive represents the pathologic results of LSIL + .

**Table 5:** The comparison of clinical colposcopists and the classification model.

	Sensitivity	Specificity	Accuracy	PPV	NPV
Expert1	61.40%	84.31%	75.38%	71.43%	77.37%
Expert2	68.87%	75%	72.84%	59.96%	81.58%
Expert3	50.47%	70.34%	71.78%	57.91%	83.36%
Expert4	75%	63.32%	67.88%	56.72%	79.80%
Expert5	70%	43.48%	51.51%	35%	76.92%
Average of experts	70%	72.92%	71.83%	60.61%	80.33%
Results in ordinary images	85.38%	82.62%	84.10%	85.02%	83.03%
Results in high definition images	73.37%	58.16%	63.83%	51.07%	78.58%

**Figure 7:**
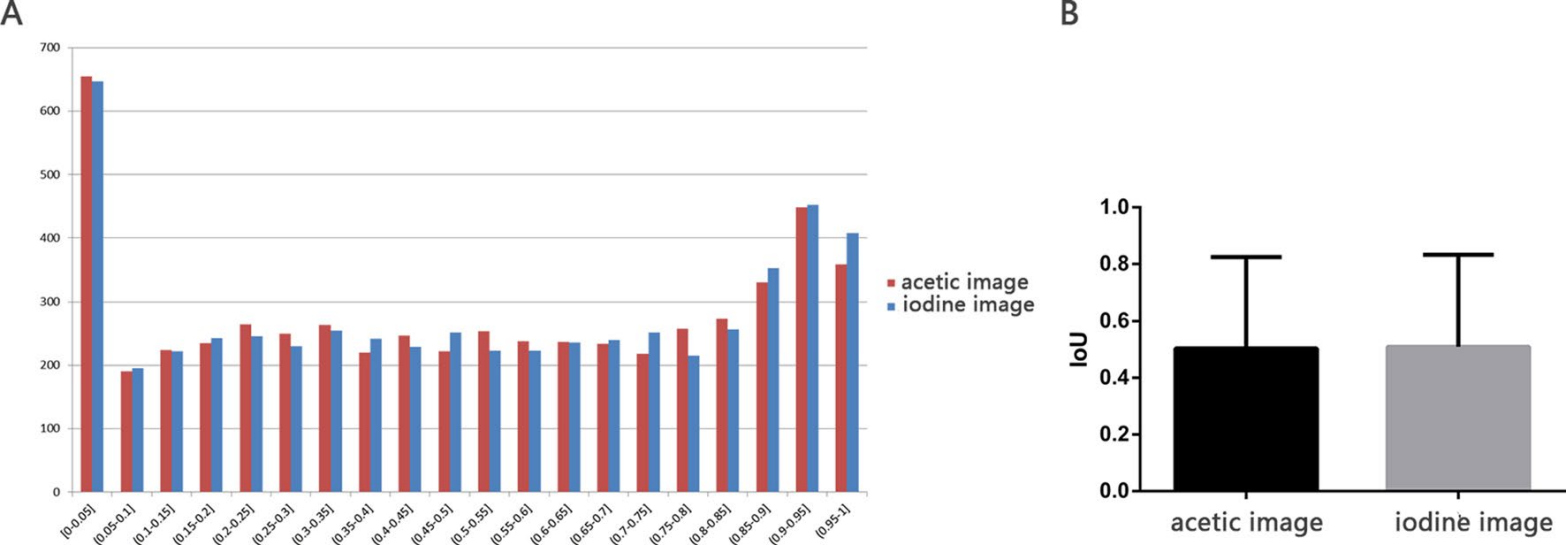
(**A**) The distribution of IoU in detection model in high-definition images. (**B**) The mean IoU in detection model in high-definition images.

In the segmentation model, the total accuracy, normal recall and SIL recall were 94.32%, 96.84%, 85.35% in the high-definition acetic images and 94.52%, 94.92%, 85.87% in the high-definition iodine images. The detection model, detected 84.76% and 82.61% HSIL regions in high-definition acetic images and iodine images, respectively, with a PPV for HSIL of 20.62% and 20.56%. The distribution of IoU and mean IoU of high-definition images were shown in Fig. 7. Moreover, the model could “hit” 691 cases through high-definition acetic images and 685 cases through high-definition iodine images among 763 HSIL cases. The prediction sensitivity at the patient level reached 90.56% in acetic images and 89.78% in iodine images. In Table 6, experts presented a higher HSIL biopsy accuracy of 22.22–30.57% than the detection model in ordinary images and high definition images. Compared to colposcopists, the biopsy number taken in each case using the detection model was slightly higher (2.79 vs. 2.39).

**Table 6:** The accuracy of colposcopy-guided biopsy by colposcopists and the accuracy of detection model.

	HSIL accuracy	SIL accuracy	Average biopsy number per case
Expert1	25.11%	66.01%	2.49
Expert2	24.35%	66.88%	2.36
Expert3	29.89%	68.38%	2.31
Expert4	30.57%	70.64%	2.42
Expert5	22.22%	66.67%	2.7
Average of experts	27.5%	67.97%	2.39
Results in ordinary images	21.22%	64.41%	2.79
Results in high definition images	20.62%	48.12%	2.63

## Discussion

Studies of deep learning in colposcopy images are quite limited, and most of them focused only on the classification of the acetic images collected from the existing database. Tao Xu et al.^25^ combined the pap test result, HPV test result, age, PH value and the cervicography observation result with the acetic image to output the result and gained a sensitivity of 87.83% and a specificity of 90%. However, this kind of algorithm could only be realized on the basis of an accurate cervicography observation result, representing less clinical value. A research study in Germany enrolled 198 women who had received colposcopy examination and biopsied and extracted 211 CIN1 annotations and 164 CIN2+ annotations. The deep learning model reached an accuracy of 80%, a sensitivity of 85% and a specificity of 75%^26^. A research study in Japan^27^ collected 485 colposcopy images and divided them into three categories of atypia, carcinoma in situ and invasive cervical cancer. The final accuracy of the deep learning model was 50%, which was higher than the accuracy of 33% in random classification. The research also demonstrated that the classification of CIN2+ with CIN1 was more significant in clinical practice. Another study collected 330 patients who received colposcopy guided biopsy to train a CNN model to identify HSIL images. The sensitivity, specificity and accuracy were 82.3%,79.7% and 80.0% , respectively, with an AUC of 0.826^28^.

All the above studies enrolled a limited number of colposcopy cases and focused only on the classification task. Our classification model gained a sensitivity of 85.38% with an acceptable specificity of 82.62%, which performed better than the above studies. In addition, with an AUC of 0.9261, the classification model we established qualified for primary triage in colposcopy.

Research on the accuracy of clinical experts in colposcopy varies greatly. Prajakta Adsul et al. calculated the colposcopy diagnosis and the biopsy pathologic results in 1,482 subjects and found that the agreement rate of the two results was only 65.5%, and the colposcopists would always underestimate the lesions^29^. Margaret E. Baum et al. compared the diagnostic accuracy of different colposcopy operators and found that the nurse practitioners obtained the highest accuracy of 92%, and the accuracies of R2, R3 and R4 residents were 77%, 75% and 73%, respectively, with an average accuracy of 77%^30^. A meta-analysis of 86 studies demonstrated that the average sensitivity of colposcopists was 96% and the average specificity was 48%, with an AUC of 0.8 to differentiate normal and abnormal cases; the average sensitivity, specificity and AUC were 85%, 69% and 0.82 to distinguish HSIL cases from LSIL cases and normal cases, respectively^31^. In conclusion, the diagnostic accuracy of colposcopy relied greatly on the experience of operators, and the accuracy of most colposcopists remained below 80%. The classification model used in this article achieved an accuracy of 84.10% in ordinary images, which was higher than the accuracy of colposcopists in the literature and the accuracy of the five experts in Women’s Hospital.

For the high-definition images, the diagnostic accuracy was 63.83%, lower than that in ordinary images. Tracing back, we found that the ordinary images and the high-definition images had different distributions of standard deviation and variance in the image features including brightness, contrast, RGB colour, saturation and other factors we may not focus on now. The higher saturation and brighter colour in high definition images might lead to the highlight of acetic white and iodine nonstaining areas in normal cases, which might be mistaken with LSIL cases, accounting for the unsatisfactory performance of the classification model. Besides, the detection model could “hit” approximately 88% of HSIL patients in ordinary images and approximately 90% of HSIL patients in high-definition images. The better performance may also be attributed to the highlighting of lesions with high saturation and brightness.

Our study combined the multimodal classification model, segmentation model, and detection model to build a comprehensive system to cope with colposcopy images and to assist diagnosis and biopsy for HSIL for the first time. The ordinary images enrolled in the study were shot by three main colposcope brands, including the electronic colposcope and the photoelectric colposcope. All three types of images presented perfect receptivity to our models. Besides, in the high definition images shot by another two electronic colposcopes, the models we established could also reach the diagnostic accuracy equal to the junior experts and presented better ability to detect HSIL.

Nevertheless, more investigations need to be carried out in the future. A large number of images from colposcopes of various brands, especially high-definition images, were required to improve the existing models. A prospective, large-scale, multicentre clinical trial needs to be carried out to evaluate the clinical value.

## Methods

### Data resource

**Figure 8:**
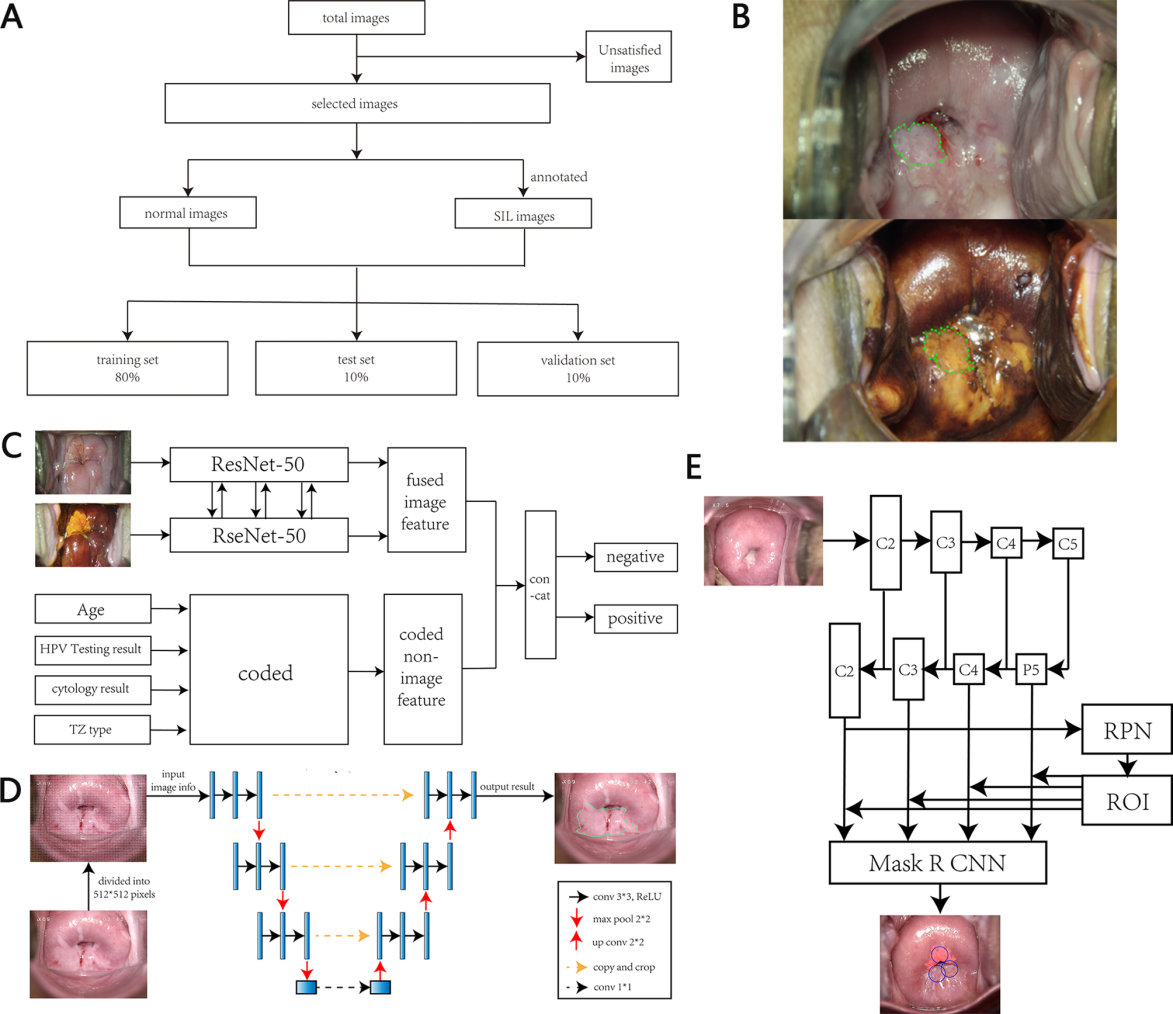
(**A**) The flowchart of case collection. (**B**) The representative acetic image and iodine image after annotation. (**C**) The diagram of the classification model. (**D**) The diagram of the segmentation model. (**E**) The diagram of the detection model.

All the colposcopy images of the modeling dataset were collected in women’s hospital, school of medicine, Zhejiang University from August 2013 to March 2019. Those who met the following conditions were excluded: without complete clinical and pathological information (age, result of HPV testing and cytology); without biopsies; pathologically diagnosed as invasive cervical cancer or glandular intraepithelial lesions; with poor-quality colposcopy images (blur, over-reflection, incomplete cervix exposure, severe bleeding, lesions covered by vaginal discharge). For each qualified case, her colposcopy images including saline image, acetic image and iodine image at the magnification of 7.5 were collected, as well as the corresponding clinical data including patient’s age, results of HPV testing and cytology, type of transformation zone (TZ),and pathologic diagnosis. All the colposcopy images were from ordinary electronic colposcope and photoelectric integrated colposcope (hereafter called ordinary images).The flowchart of case collection was shown in Fig. 8A. The research was approved by the Medical Ethics Committee of Women’s Hospital, School of Medicine, Zhejiang University, and written informed consent was obtained from all subjects. All the methods were performed in accordance with the relevant guidelines and regulations.

Women with different ages may manifest different degrees of reliability in HPV infection status and colposcopy impression. They are also applied to different screening strategies^32^. Thus, the ages of the patients were divided into three groups for better management (Table 7).

**Table 7:** The coding method of age.

Age	Meaning	Code
Group A	The age of the patient is between 20 and 25,including 20	Yes is marked as 1, otherwise as 0
Group B	The age of the patient is between 25 and 55,including 25	Yes is marked as 1, otherwise as 0
Group C	The age of the patient is between 55 and 66,including 55	Yes is marked as 1, otherwise as 0

HPV testing was performed by food and drug administration (FDA) approved techniques, including Hybrid Capture 2 (HC2) hr-HPV DNA Test^33^ (Qiagen, Gaithersburg, MD), Cobas 4,800 Test^34^ (Roche Molecular system Inc, Pleasanton, CA), Cervista HPV HR Test^35^ (Hologic Inc., Madison, WI), and Aptima HPV Assay^36^ (Hologic Gen-Probe Inc., San Diego, CA).

The results of HPV testing were divided into negative and positive (Table 8).

**Table 8:** The coding method of HPV result.

HPV result	Meaning	Code
HPV negative	HPV negative	HPV negative is marked as 1, otherwise as 0
HPV positive	High Risk HPV positive using whichever methods mentioned above	HPV positive is marked as 1, otherwise as 0

The cytology diagnoses were divided into six categories, according to the Bethesda 2014 classification ^37^ (Table 9).

**Table 9:** The coding method of cytology result.

TCT result	Meaning	Code
NILM	Negative for Intraepithelial Lesion or Malignancy	Yes is marked as 1, otherwise as 0
ASCUS	Atypical Squamous Cells of Undetermined Significance	Yes is marked as 1, otherwise as 0
LSIL	Low-grade Squamous Intraepithelial Lesion	Yes is marked as 1, otherwise as 0
ASC-H	Atypical Squamous Cells- cannot exclude a High-grade lesion	Yes is marked as 1, otherwise as 0
HSIL	High-grade Squamous Intraepithelial Lesion	Yes is marked as 1, otherwise as 0
SCC	Squamous Cell Carcinomas	Yes is marked as 1, otherwise as 0

TZ types were divided into three categories according to the International Federation for Cervical Pathology and Colposcopy (IFCPC) colposcopy terminology^38^ (Table 10).

**Table 10:** The coding method of TZ type.

Age	Meaning	Code
Type 1 TZ	The squamous columnar junction can be fully visualized without the help of equipment	Yes is marked as 1, otherwise as 0
Type 2 TZ	The squamous columnar junction can be fully visualized with the help of equipment	Yes is marked as 1, otherwise as 0
Type 3 TZ	The squamous columnar junction cannot be fully visualized, even with the help of equipment	Yes is marked as 1, otherwise as 0

The pathological diagnoses were divided into normal, low-grade squamous intraepithelial lesion (LSIL, including the condylomatous variant), high-grade squamous intraepithelial lesion (HSIL) based on the 2014 World Health Organization (WHO) Classification of Tumors of the Female Genital Tract^39^.

The independent validation dataset of colposcopy images and the corresponding age, HPV testing results, cytology results, type of transformation zone and pathologic diagnosis were collected in order to better evaluate the established models. All cases were collected in women’s hospital, school of medicine, Zhejiang University from March 1st, 2019 to September 12nd 2019. And all colposcopy images were from high definition electronic colposcope (hereafter called high definition images). The exclusion criterions were the same as above.

The retrospective study was approved by the Medical Ethical Committee of Women’s Hospital, School of Medicine, Zhejiang University. (No. 20180059).

### Data pre-process

One acetic image and one iodine image of each qualified case were kept and resized into 512*512 pixels. All the resized images of the modeling dataset were divided into 100 categories by K-means algorithm and randomly relocated into three sets of the training set, the valid set and the test set with the ratio of 8:1:1. Normal images, LSIL images and HSIL images were relocated separately so that they would be equally distributed into the three sets. Only the valid set was calculated to evaluate the performance of models. Pathologically diagnosed lesions were annotated by labelme^40^ software in every acetic image and every iodine image (Fig. 8B).

Text information including age, results of HPV testing and cytology, and TZ type were coded by the methods represented in Tables 1 to 4. As an example, a 45 year-old patient with HR-HPV positive and ASCUS cytology result, type 3 TZ, her texting code is 01001010000001.

### Transfer learning model

In order to get high efficiency, a pretrained deep learning model was obtained by training a ResNet^41^ model from a database called ImageNet, which contains more than 1 million images of over 1,000 categories. On that basis, colposcopy images were input to fine-tune multi-modal ResNet classification model, U-Net^42^ segmentation model and Mask R-CNN^43^ detection model, which use the pre-trained ResNet model as backbone.

### Multi-modal ResNet classification model to simply classify the images into two groups

Two ResNet-50 models were used for acetic image and iodine image, respectively. Cervix regions were firstly extracted due to other undesired information on the acetic and iodine images such as text, equipment and non-cervix tissues. Since clinical diagnosis were often made after a long comparison of the acetic and the iodine images, fusing the acetic image features and iodine image features during the training process can better capture cervical lesions and to offer a more scientific diagnosis. In the end, the coded non-image information of age, HPV testing result, cytology result and TZ type were input into the model and integrated with the fused image features. All the images will be classified into two groups: the negative group which means no squamous intraepithelial lesion (SIL, including LSIL and HSIL) in the cervix and the positive group which means one or more SIL were found in the cervix (Fig. 8C).

For classification model, the input image was scaled to 512 on the shorter edge. We used BCE loss with positive weith of 10. Batch size was set to 16. SGD optimizer was used with learning rate 1e−4, weight decay 1e−4 and momentum 0.9. Learning rate was multiplied with 0.9 when training loss was no longer reduced during 10 epochs.

### U-Net segmentation model to segment the lesions apart from the normal areas

Same as the classification model, the U-Net model was also fine-tuned on the basis of the transfer-learning ResNet model. The colposcopy images were resized to 512*512 pixels, and each pixel was labeled as “1” for “lesion” or “0” for “normal” according to the annotations made by the colposcopy experts. In the end, all the lesions will be highlighted, representing the possible biopsy sites (Fig. 8D).

Taking every pixel as the object, recall and dice were calculated to evaluate the two models using the following formula: Recall = true positive pixels/ predicted positive pixels. DICE = 2*true predicted pixels/(predicted positive pixels + true positive pixels).

For segmentation model, the input image was scaled to 512 on the shorter edge. Focal loss was used. Batch size was set to 8. SGD optimizer was used with learning rate 1e-2, weight decay 1e-4 and momentum 0.9. Learning rate was multiplied with 0.9 when training loss was no longer reduced during 10 epochs.

### Mask-R-CNN detection model to offer the final HSIL biopsy sites

Based on the transfer-learning ResNet model, the Mask R-CNN model detected lesion regions on colposcopy images according to the ground truth of delineating bounding boxes on existing segmentation annotation. Compared to the corresponding ground truth bounding boxes, the predicted ones offered by the detection model were considered positive when their Intersection over Union (IoU) value is more than 0.5. The loU is defined as the area of the intersection divided by the area of the union of a predicted bounding box (B_p_) and a ground truth box (B_gt_): loU = area (B_p_ ∩ B_gt_)/area (B_p_ ∪ B_gt_). In order to decrease the biopsy number, the model chose only the top 3 possible HSIL predicted bounding boxes in the premise of acceptable accuracy (Fig. 8E).

For detection model, the input image was scaled to 600 on the shorter edge. Batch size was set to 4. SGD optimizer was used with learning rate 2e−3, weight decay 1e−4 and momentum 0.9. We trained the model for 80 k iteration and learning rate was divided by 10 on 50 k, 70 k iteration.

For all the three models, we applied random color, random contrast, random saturation, and random hue transformation.

## Supplementary information

Supplementary file1 (DOCX 25 kb)

Supplementary file2 (TIF 5033 kb)

Supplementary file3 (TIF 5952 kb)
