# Supplementary material for: The application of deep learning based diagnostic system to cervical squamous intraepithelial lesions recognition in colposcopy images
Source: Sci Rep. 2020 Jul 15;10:11639. doi: 10.1038/s41598-020-68252-3 (PMC7363819; doi:10.1038/s41598-020-68252-3)
Supplement: Supplementary file 1 — Supplementary file1 (DOCX 25 kb) [file 41598_2020_68252_MOESM1_ESM.docx]

**The Application of Deep Learning Based Diagnostic System to Cervical Squamous Intraepithelial Lesions recognition in Colposcopy Images**

Chunnv Yuan^1┽^, Yeli Yao^2┽^, Bei Cheng^2^, Yifan Cheng^2^, Ying Li^2^, Yang Li^2^, Xuechen Liu^4^,Xiaodong Cheng^2^, Xing Xie^2^, Jian Wu^4^, Xinyu Wang^2,3^, Weiguo Lu^2,3*^

*Correspondence: Weiguo Lu (lbwg@zju.edu.cn)

^┽^：Chunnv Yuan and Yeli Yao contributed equally to this work.

1 Women’s Reproductive Health Laboratory of Zhejiang Province, Women’s Hospital, School of Medicine, Zhejiang University, Hangzhou 310006 Zhejiang, China

2 Department of Gynecologic Oncology, Women’s Hospital, School of Medicine, Zhejiang University, Hangzhou, 310006, China.

3 Center for Uterine Cancer Diagnosis & Therapy Research of Zhejiang Province, Hangzhou, 310006, China.

4 College of Computer Science and Technology, Zhejiang University, Hangzhou, 310027, China.

Supplementary legend:

Figure S1. The representative failed images of the U-Net segmentation model.

1. The lesion in the direction of twelve o’clock was missed by the model.
2. The lesion lying on the upper lip of the cervix was missed by the model.
3. Two lesions in the direction of one o’clock and ten o’clock were missed, and the lesion in the direction of six o’clock was enlarged by the model.
4. The lesion in the direction of ten o’clock was missed by the model.
5. Tesion in the direction of twelve o’clock was missed by the model.
6. The lesion below in the direction of far five o’clock was misdiagnosed by the model.

Figure S2. The representative failed images of the Mask R-CNN detection model.

1. Lesions in the direction of two o’clock and four o’clock were misdiagnosed as SIL by the model (with the red area indicating real lesion).
2. The lesion in the lower lip of the cervix was misdiagnosed as SIL by the model (with the red area indicating real lesion).
