# Supplementary figures and images for: The application of deep learning based diagnostic system to cervical squamous intraepithelial lesions recognition in colposcopy images
Source: Sci Rep. 2020 Jul 15;10:11639. doi: 10.1038/s41598-020-68252-3 (PMC7363819; doi:10.1038/s41598-020-68252-3)

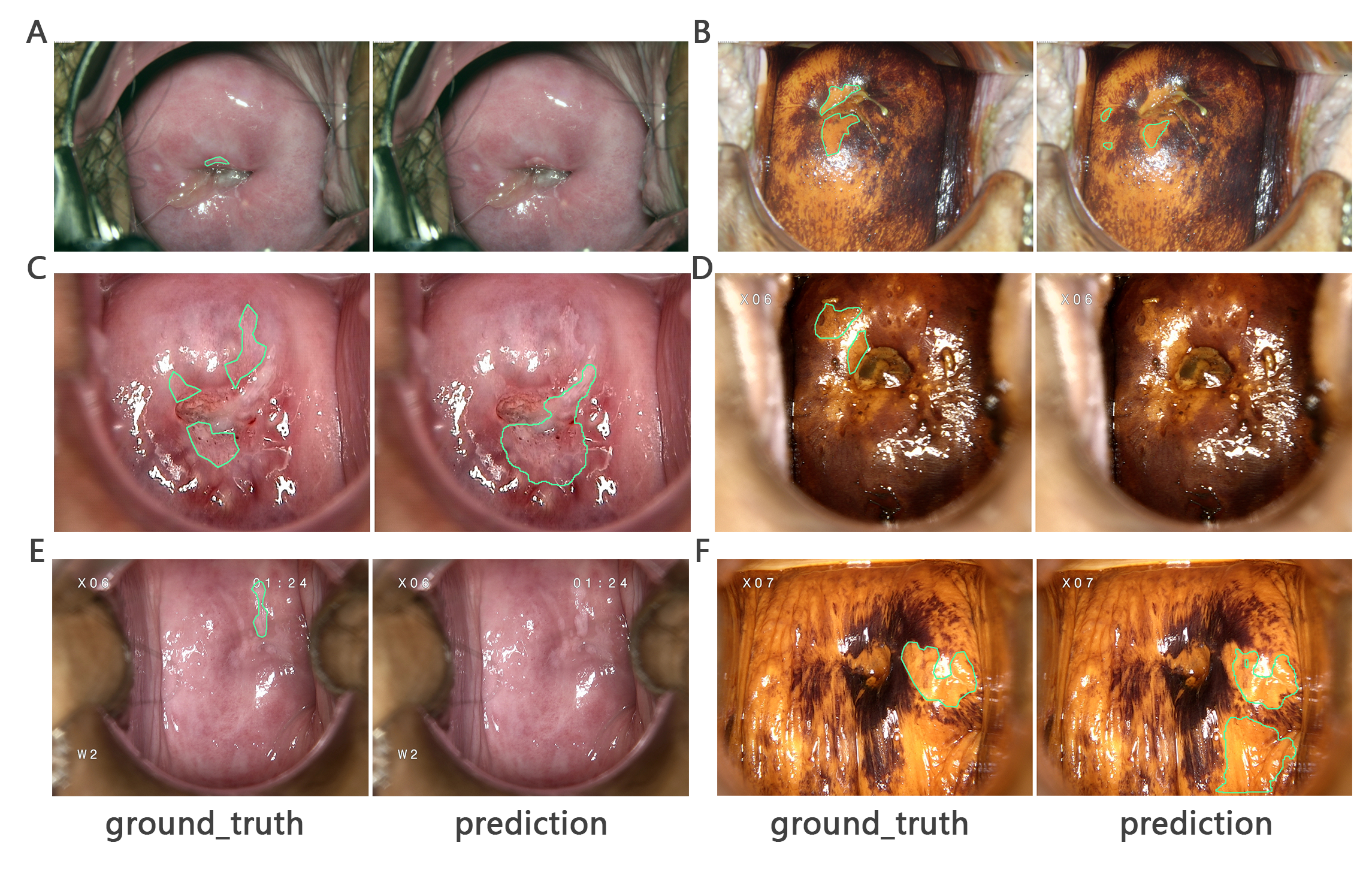

Supplement: Supplementary file 2 — Supplementary file2 (TIF 5033 kb) [file 41598_2020_68252_MOESM2_ESM.tif]

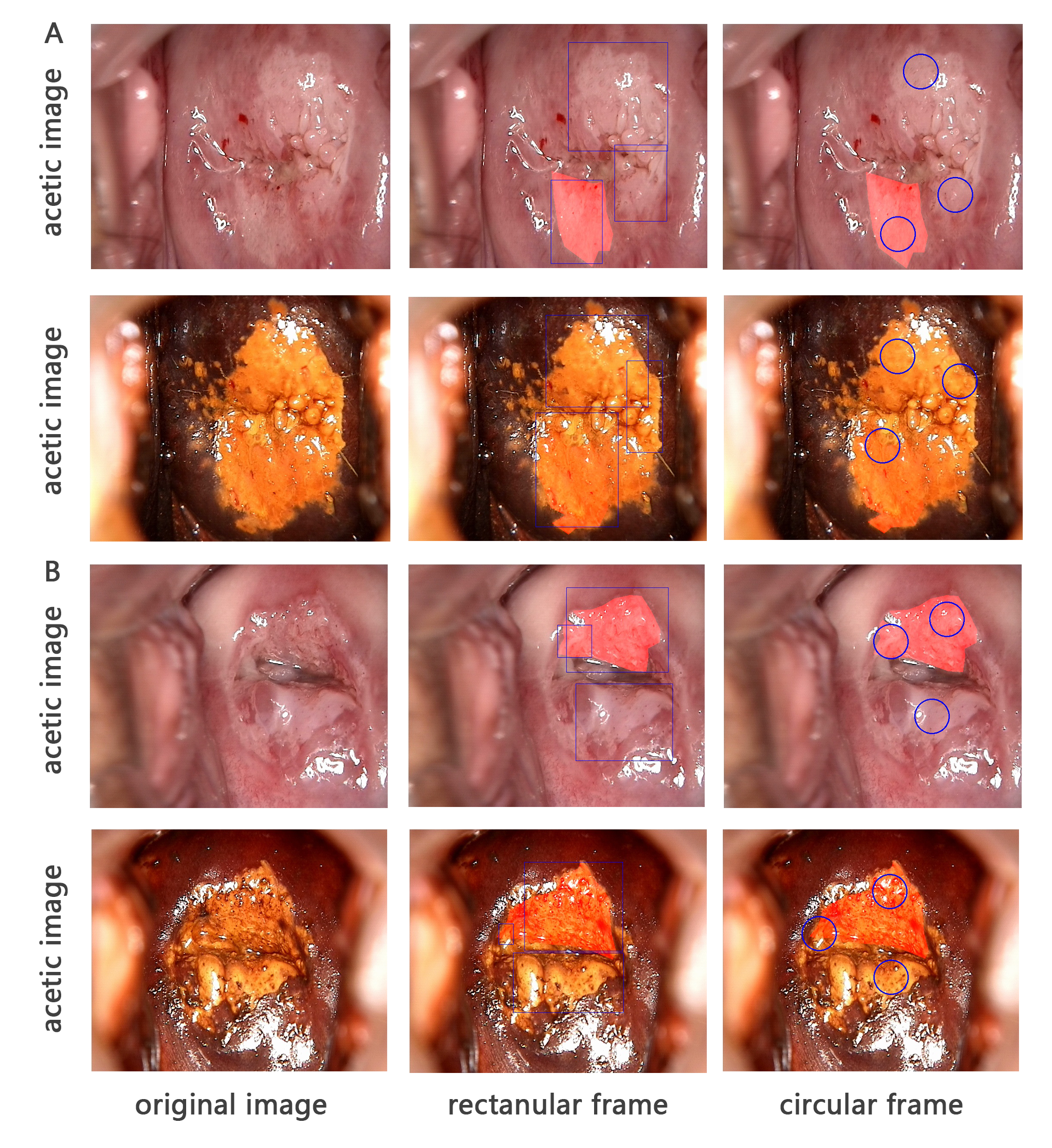

Supplement: Supplementary file 3 — Supplementary file3 (TIF 5952 kb) [file 41598_2020_68252_MOESM3_ESM.tif]
